# A comparison of case-only designs for detecting gene × gene interaction in rheumatoid arthritis using genome-wide case-control data in Genetic Analysis Workshop 16

**DOI:** 10.1186/1753-6561-3-s7-s73

**Published:** 2009-12-15

**Authors:** Geraldine M Clarke, Fredrik H Pettersson, Andrew P Morris

**Affiliations:** 1 Wellcome Trust Centre for Human Genetics, University of Oxford, Roosevelt Drive, Oxford, OX3 7BN, UK

## Abstract

We compare and contrast case-only designs for detecting gene × gene (G × G) interaction in rheumatoid arthritis (RA) using the genome-wide data provided by Genetic Analysis Workshop 16 Problem 1. Logistic as well as novel multinomial and proportional odds models that do not depend on the specification of additive or dominant models for susceptibility loci were applied to the case-only sample. We identified 519 significant interactions (*p *< 1 × 10^-4 ^in at least one test). All methods detected unique significant interactions; 169 were common to more than one model and only 21 were common to all models. Results emphasize that categorization of the genetic variables and choice of regression model are critical and hugely influential in the identification of G × G. Porportional odds and multinomial methods provide new tools for identification of G × G interactions.

## Background

Various strategies have been proposed to test hypotheses related to gene × gene (G × G) interaction in case-control data. G × G interaction occurs when the effect of one gene in determining the occurrence of disease is modified by the presence or absence of another gene. Strategies for detection can involve utilization of the whole sample or just the cases, and associated tests are derived theoretically on the basis of underlying models of disease penetrance. The power of a test to detect an interaction depends on the size of the detectable effect, the sample size and composition, and the suitability of the test as it relates to the true underlying model. In this study, we seek to compare and contrast how association findings can vary as a result of the different regression models applied to detect G × G interaction in the case-only sample.

Motivated by differences in the magnitude of genetic effects associated with rheumatoid arthritis (RA) observed at genes *PTPN22*, *CTLA4*, and *PADI4 *across samples of common ancestry [1], we concentrate on interactions between each of these genes and a genome-wide subset of markers selected to be in approximate linkage equilibrium using the genome-wide data provided by Genetic Analysis Workshop 16 (GAW16) Problem 1. Specifically we propose to compare case-only designs that test for single-nucleotide polymorphism (SNP)-by-SNP interactions in RA between alleles at loci in candidate genes *PTPN22*, *PADI4*, and *CTLA4*, each known to have a previous putative marginal association with RA, and alleles at a selected subset of markers in the GAW16 data from the North American Rheumatoid Arthritis Consortium (NARAC).

Assuming that the genes being studied are not in linkage disequilibrium, case-only designs are a valid approach for the detection of G × G interaction and provide increased statistical efficiency over case-control analyses [2]. Yang et al. demonstrated their results assuming binary genotype variables; here we consider case-only designs that allow for disease susceptibility genes with multiple genetic variants.

## Methods

### Materials

The data set for these interaction studies of RA were provided as part of GAW16 Problem 1. The case-control data set included 868 cases and 1194 controls genotyped with the Illumina 550 k chip (531,689 SNPs). All samples were retained after checks for contamination and relatedness. 496,578 SNPs (93.4%) passed our quality control filters. Of these, 21,959 have a study-wide minor-allele frequency (MAF) less than 1% and were excluded from the analysis. Of the remaining 447,619 SNPs, 6 were on *PTPN22*, 7 were on *PADI4*, and 2 were on *CTLA4*; these 17 SNPs in candidate genes are referred to as the gene SNPs. A subset of 81,596 SNPs with pairwise linkage equilibrium *r*^2 ^< 0.2 was created by considering all pairs of retained SNPS in sliding windows of size 50; these SNPS are referred to as the equilibrium SNPs. Additional phenotype data including sex, shared epitope alleles, anti-cyclic citrullinated peptide (CCP) and rheumatoid factor were available for both cases and controls.

### Models

We consider a binary trait that is influenced by two bi-allelic disease susceptibility loci *F *and *G *according to a model of joint locus effects. Here we assume *F *denotes a candidate gene SNP and *G *denotes an equilibrium SNP. We test for G × G interaction between gene and equilibrium SNPs using tests based on logistic, proportional odds, and multinomial generalized linear regression models. For each model, there are two regressions: first *F *is modelled as the outcome variable and *G *the predictor, then vice versa. The outcome variable is categorized appropriately according to the relevant model: a binary categorization for the logistic model, an ordinal categorization for the proportional odds model, and a nominal categorization for the multinomial model. The predictor variable is categorized as an ordinal variable in all the regressions. Table 1 summarizes the generalized linear regression models considered. Each model generates a likelihood and G × G test of interaction are based on standard likelihood ratio (LR) statistics, which compare the likelihood under the null hypothesis of no interaction, where the coefficients associated with the predictors are constrained to be zero, to the likelihood under the alternative hypothesis, where those coefficients are free. All regressions were further adjusted for sex. In total, 17 (candidate gene SNPs) × 81,596 (SNPs in linkage equilibrium) × 6 (regressions) = 8,322,792 tests were performed.

**Table 1:** Generalized linear regression models used for testing G × G interactions

Model	Regression^a^
Multinomial	
	

Proportional odds	
	

Logistic	
	

^a^*F *= 0,1,2 denotes the number of risk alleles at locus *F*. *FDOM *denotes a dummy variable taking the value 1 if *F *= 1 or *F *= 2 and zero otherwise. Variables *G *and *GDOM *are defined similarly for alleles at locus *G*.

## Results

**Figure 1:**
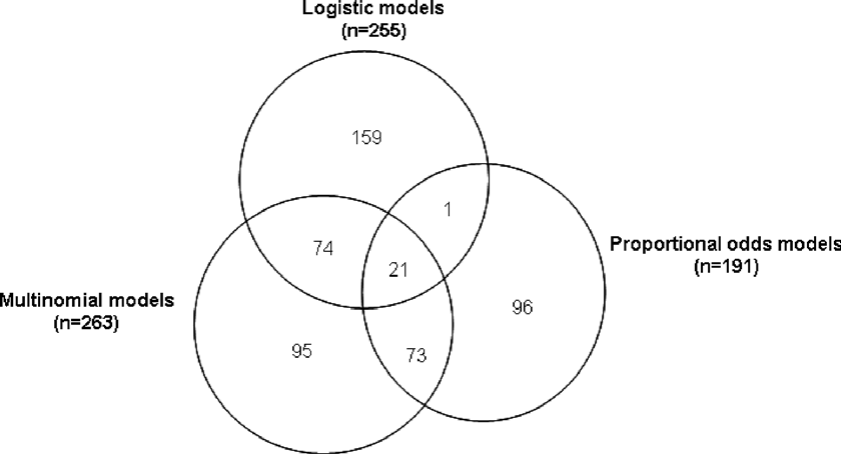
**Summary of significant interactions**. Summary of significant interactions detected according to regression models applied. An interaction is included in a method if the *p*-value in that method is <1 × 10^-4^.

No pairs of markers in any of the tests performed were found to interact at genome-wide significance under a conservative threshold that accounted for the number of tests performed. However, 519 unique pairs of markers were found to have significant interactions (*p *< 1 × 10^-4^) in at least one test performed. Figure 1 illustrates counts of these pairs of markers according to the method or model under which they were detected.

The logistic models detected 255 significant interactions: 159 unique, 95 in common with multinomial models, and just 22 in common with proportional odds models. Proportional odds models detected 191 interactions: 96 unique and 94 in common with multinomial models. Almost all (174 of 191) of the interactions detected by proportional odds models indicated no justification for the additional degree of freedom afforded by the multinomial models (*p *< 1 × 10^-5^). The multinomial models detected the most (263) interactions: 95 of the 263 were uniquely detected. The logistic models showed the most dependence on choice of SNP as response variable and the proportional odds, the least. Only 1% (2 of 255) of the significant interactions detected in the logistic regressions were detected independently of which SNP was selected for the response compared to 51% (98 of 191) in the proportional odds regressions and 20% (53 of 263) in the multinomial regressions. Genomic control inflation factors [3] calculated for each gene SNP and regression were consistently close to one, suggesting no adjustment was required for residual population structure at the particular set of genes studied here.

We reviewed the nine pairs of markers with the most significant interactions (*p *< 1 × 10^-7 ^in at least one model). Of interest are an interaction between rs6683201 on *PADI4 *and rs2899664 on chromosome 15 at 58.99 Mb in retinoic acid receptor-related orphan receptor (*RORα*) and between rs733618 on *CTLA4 *and rs2241351 on chromosome 19 at 18.29 Mb in gastrin-releasing peptide (*GRP*, or *LSM4*). *RORα *plays a function in bone metabolism. Levels of *GRP *in blood and synovial fluid correlate with levels of pro-inflamatory cytokines in patients with RA.

## Conclusion

When comparing the imposition of binary constraints on the genetic locus selected as the response variable in the logistic models with the additive constraint imposed on the genetic locus selected as predictor, the logistic models show the most dependence on choice of SNP as response variable. Similar findings hold for the multinomial models, with their nominal constraint on the response variable compared with the additive constraint on the predictor variable. The proportional odds models are less dependent on choice of SNP on the response variable because the proportional odds assumption imposed on the response variable suggests a trend with increasing risk alleles more in tune with the additive constraint imposed on the predictor variable. Clearly, the selection of a binary constraint on the predictor in the logistic models, or a nominal constraint in the multinomial models, would reduce the dependence. These results emphasize that selecting an appropriate inheritance pattern at each genetic locus is critical to the success of G × G interaction studies.

The majority of significant interactions were uniquely detected in a single regression model. Moreover, 264 of the 519 interactions detected were not detected by the most commonly used logistic models. Logistic models *require *an assumption of autosomal dominant inheritance at one of the genetic loci under consideration in order to ensure the resulting response variable is binary. When the inheritance pattern is actually recessive, the logistic models is ill-suited to detect an interaction. Multinomial models are most appropriate when there is significant deviation from additivity (either positive, indicating a dominant effect, or negative, indicating a recessive effect) at the genetic locus assigned as the response variable. The proportional odds models is most appropriate when there is no or just modest deviation from additivity at the genetic locus assigned as the response variable [4]. These results further confirm that the choice of and justification of the assumptions underlying the selection of appropriate models to robustly detect G × G interactions is critical.

Under the null hypothesis of no interaction, none of the tests show an increase in the type I error rate, while under the alternative all tests have power greater than the type I error rate, suggesting that all tests are valid tests of G × G interaction. In general, however, there are several limitations of using case-only designs to measure G × G interaction. First, independence between interacting genes is critical to the validity of case-only estimates of G × G interaction; specifically, the genes under study must be in linkage equilibrium and have independent gene frequencies in the population under study. Genes on different chromosomes are unlikely to be correlated and interpretation of interactions found between genes on the same chromosome using case-only designs will need to be interpreted cautiously. To ensure independence, we excluded comparisons between pairs of markers less than 1 Mb apart from our analysis. Second, the case-only design measures statistical rather than biological interaction; biological interaction is arguably more relevant [5]. Third, case-only designs cannot estimate risks associated with each gene alone; other types of studies are required to assess these risks [6,7]. Finally, population stratification may bias results in case-only designs of G × G interaction when the allele frequencies at the SNPs under study differ between the underlying sub-populations. Genomic control inflation factors suggested no significant residual population structure in the set of gene SNPs studied here.

More work is required to further assess the impact of these limitations in each of the case-only designs considered here. Specifically, work is required to interpret the regression coefficients with a view to estimating both the size and the precise nature of the interactions detected and to examine in greater detail the relative effects of population stratification in each of these case-only designs. More work is also required to compare the power of each model to detect interactions under different underlying models of penetrance. However, these preliminary findings suggest that consideration of mltinomial and proportional odds regression models are viable alternatives to logistic models in case-only designs of G × G interaction.

## List of abbreviations used

G × G: Gene × gene; GAW16: Genetic Analysis Workshop 16; LR: Likelihood ratio; MAF: Minor-allele frequency; RA: Rheumatoid arthritis; SNP: Single-nucleotide polymorphism

## Competing interests

The authors declare that they have no competing interests.

## Authors' contributions

GMC, FHP, and APM conceived of the study. GMC and APM participated in its design and coordination. GMC performed the statistical analysis and drafted the manuscript.
